# Upper gastrointestinal bleeding due to Dieulafoy’s lesion of the stomach: a rare case report

**DOI:** 10.17179/excli2023-6407

**Published:** 2023-08-17

**Authors:** Alsalt AL-Busaidi, Jaifar Alomairi, Omer Alabri, Eissa Alwheibi, Alazhar Almaghadari, Mhmod R. Kadom, P. Ronan O'Connell

**Affiliations:** 1 School of Medicine, Royal College of Surgeons in Ireland, Dublin, IRL; 2 Surgery, University College Dublin, Dublin, IRL

**Keywords:** Dieulafoy's lesion, exulceratio simplex, upper gastrointestinal bleeding, hematemesis, pulsatile bleeding

## Abstract

Dieulafoy's lesion is a life-threatening and rare vascular malformation of the submucosal vessel that protrudes to the mucosa of the gastrointestinal tract. The vessel is abnormally dilated, and if it ruptures, it can cause severe acute gastrointestinal bleeding. We report an upper GI bleeding case due to Dieulafoy's lesion in the gastric fundus of the stomach in a 76-year-old female. The patient presented with hematemesis and melena associated with anemia. An esophagogastroduodenoscopy (OGD) was performed which showed profuse pulsatile bleeding at the gastric fundus. Following that, gastrotomy confirmed the diagnosis of Dieulafoy's lesion. Endoscopy is the main diagnostic and therapeutic tool for Dieulafoy's lesion. Endoscopic treatment includes injective, ablative and mechanical therapies. The majority of cases are treated endoscopically, while in some cases, surgical intervention is deemed to be necessary as it is currently the only definitive treatment of Dieulafoy's lesion.

## Introduction

Dieulafoy's lesion, also termed 'calibre persistent artery', is an extremely rare developmental vascular malformation and an important cause of acute gastrointestinal bleeding which tends to be severe, recurrent, and life-threatening. It is mostly associated with upper and middle gastrointestinal bleeding, yet in rare instances, can cause lower gastrointestinal hemorrhage. Such lesions were first described by the French surgeon Paul Georges Dieulafoy in 1989 as “exulceratio simplex” (Nojkov and Cappell, 2015[15]).

Dieulafoy's lesions occur not because of a defect in the vascular wall, but rather because the submucosal vessel is abnormally dilated with a diameter of 1 to 3 mm as it runs close to the mucosa of the gastrointestinal tract (GIT). The protrusion of the vessel to the mucosa causes a minute mucosal defect with fibrinoid necrosis at its base. These lesions are characterized by bleeding in the absence of macroscopic ulceration or erosion. Dieulafoy's lesions can occur anywhere in the GIT, although predominantly are found in the gastric fundus, usually along the lesser curvature (Chung et al., 2000[7]; Malik and Anjum, 2023[14]).

The etiology behind Dieulafoy's lesion is unknown, however, bleeding is often associated with comorbidities such as cardiovascular disease, hypertension, chronic kidney disease, and the use of NSAIDs (Rockey, 2022[18]). Patients are typically asymptomatic before presenting with acute gastrointestinal bleeding, which may manifest as hematemesis, melena, or hematochezia. 

Once the site of bleeding is identified, given advances in endoscopic and interventional radiologic techniques hemorrhage can usually be controlled without recourse to surgery (Nojkov and Cappell, 2015[15]; Ribeiro et al., 2021[17]).

We present a case of upper gastrointestinal bleeding due to Dieulafoy's lesion in the gastric fundus of the stomach in a 76-year-old woman.

## Case Presentation

A 76-year-old female presented to the hospital in 2000 for left leg ulcer debridement and split-thickness skin graft (SSG) with a history of comorbidities. The patient had a history of atrial fibrillation, left ventricular failure, and chronic obstructive airway disease (COAD). She was under high-dose antiplatelet therapy with aspirin (300 mg), oral Digoxin (0.125 mg once daily), Captopril (50 mg twice daily), and Verapamil (240 mg once daily) for her cardiovascular morbidities and oral Aminophylline (225 mg twice daily) with Ipratropium Bromide/Salbutamol and Beclomethasone Inhalers for her COAD. 

The patient was found to be anemic with hemoglobin of 9.2 gm/dl, but was otherwise stable. She had hematemesis and hence was started on a high-dose Proton Pump Inhibitor (PPI). Two days later, the patient's hemoglobin dropped to 7.2 Hgb g/dl with hematemesis and melena as presenting symptoms. In addition to a high-dose PPI, six units of Red Blood Cell Count (RCC) were given over 72 hours. Over the next week, the patient's hemoglobin dropped to 6.4 Hgb g/dl and became hemodynamically unstable which required a further six RCC units. After four days, the patient presented with melena, requiring additional five units of RCC. She had to receive several more RCC units as her hemoglobin levels kept fluctuating over the next three days. The patient was successfully resuscitated after two unexpected episodes of ventricular fibrillation.

**Figure 1:**
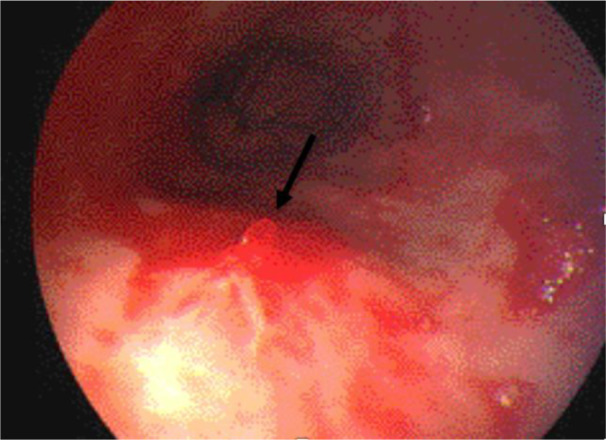
Esophagogastroduodenoscopy (OGD) showing profuse pulsatile bleeding at the gastric fundus with an adherent clot (pointed by the arrow)

Several esophagogastroduodenoscopies (OGDs) were performed in the first two weeks of her admission, but they did not yield conclusive outcomes. The most relevant OGD showed a profuse pulsatile bleeding with an adherent clot at the gastric fundus (Figure 1(Fig. 1)), along with rugal gastritis and fresh and old blood at the fundus. A gastrotomy confirmed the diagnosis of a Dieulafoy's lesion within the gastric fundus, managed surgically by under sewing the lesion. Injection of epinephrine (1:10000) did not stop the bleeding. The patient recovered and was discharged five days post-operation without complication.

## Discussion

Dieulafoy's lesion has many differential diagnoses depending on the patient's overall well-being, age, and comorbidities. Angiodysplasia is one of the most important differential diagnoses due to the similar presentation of symptoms. On an angiography, angiodysplasia may be distinguished by the existence of arteriovenous shunting and vascular ectasia, as well as by a lesion's histological analysis, which indicates the presence of aberrant submucosal arteries. Other differential diagnoses include gastric antral valvular ectasia, vascular neoplasms, telangiectasis, and connective tissue disorders. These diseases could be differentiated from Dieulafoy's lesions by the presented symptoms, OGD, and angiography (Malik and Anjum, 2023[14]).

Dieulafoy's lesion typically manifests as recurrent upper gastrointestinal bleeding, initially asymptomatic, with later melena, hematemesis, and hematochezia. It primarily affects elderly patients, males more frequently, and hospitalized patients with multiple comorbidities (Jeon and Kim, 2015[10]; Hamdoun et al., 2019[9]; Aletaha et al., 2019[1]). The patient was taking 300 mg of aspirin on alternate days, which could be a potential risk factor for experiencing multiple episodes of hematemesis and melena during her management course (Hamdoun et al., 2019[9]). 

Endoscopy is the main diagnostic tool, with the use of push enteroscopy to extend the assessment of around 150 cm through the pylorus into the small intestine. However, due to the presence of blood within the lumen and the subtle mucosal abnormality, the diagnosis may require repeated endoscopy as in the present case. Endoscopic ultrasound can be useful to identify the lesion. More invasive intervention for obscure upper GI bleeding may be required, with the use of laparoscopy or laparotomy plus push enteroscopy (Malik and Anjum, 2023[14]; Rajanthran et al., 2020[16]; Levy et al., 2022[12]).

Several approaches have been indicated for the initial management of Dieulafoy's lesions. These approaches include pre-endoscopic treatments, endoscopic therapies, combination therapies, embolization, and surgery.

Endoscopy is the main diagnostic and therapeutic tool for upper GI bleeding. The indication for endoscopic therapy depends on the stigmata of recent hemorrhage (SRH). Endoscopic therapy is recommended for major SRH cases, such as active bleeding, oozing, or the visibility of a non-bleeding vessel in peptic ulcer disease. Minor SRH such as pigmented flat spot or a simple ulcer with a clean base does not require endoscopic therapy (Cappell, 2010[4]). Endoscopic therapy divides into three modalities: injection, ablative, and mechanical therapies. In individuals with peptic ulcer disease and major SRH, monotherapy decreases the risk of rebleeding to 20 %. In contrast, combination therapy is much preferred as it reduces the risk of re-bleeding to 10 %. Combination therapy consists of an injection therapy such as epinephrine followed by mechanical or ablative therapy (Cappell, 2010[4]). Single-modality endoscopic therapy was used as much as combination therapy in a study conducted on 63 patients with Dieulafoy's lesion with 92 % effectiveness as primary hemostasis (Lara et al., 2010[11]). Studies have shown that endoscopy is a highly successful therapy in terms of primary hemostasis. Twenty patients who had endoscopic treatment (95 %) had no recurrent bleeding from Dieulafoy's lesion (Baettig et al., 1993[3]).

Endoscopic injection therapies involve the injection of epinephrine, cyanoacrylate, or sclerosing agent. Epinephrine injection promotes hemostasis by inducing vasoconstriction and mechanical compression leading to blood stasis and coagulation (Nojkov and Cappell, 2015[15]). The patient experienced a recurrence of bleeding following the withdrawal of the epinephrine injection (1:10000). Yilmaz et al. (2005[19]) reported that 26 of 28 patients (92.8 %) with Dieulafoy's lesions were successfully treated with endoscopic injection sclerotherapy. Sodium tetradecyl sulphate or ethanolamine are the usual sclerosants used agents (Chaptini and Peikin, 2008[5]). Cyanoacrylate, such as Histoacryl, is an alternative agent that is safe and effective in treating and preventing recurrent bleeding from Dieulafoy's lesion (El Sayed et al., 2015[8]; Cheng et al., 2004[6]).

Endoscopic ablative therapy includes the use of thermocoagulation treatment, such as laser photocoagulation and heater probe therapy. Thermocoagulation has shown to be significantly superior to injection monotherapy (Cheng et al., 2004[6]). In this case, laser photocoagulation would have been considered if an area of angiodysplasia was identified. Also, a heater probe treatment was not available at that time and was useful only if the site of the bleeding was identified. 

Some studies have shown that mechanical therapies are more effective in treating patients with GI bleeding from Dieulafoy's lesion when compared to other endoscopic modalities (Chung et al., 2000[7]). However, endoscopic band ligation may be less ideal than clips since it might result in the perforation of tissues and may cause a bleeding ulcer when the band detaches. This is especially important in GI segments with thin walls, such as the gastric fundus, small bowel, and right colon (Nojkov and Cappell, 2015[15]). Endoscopic clips could have been an option for this patient but were not available at that time.

In cases where endoscopic treatment is unsuccessful, as in this case, surgical intervention for Dieulafoy's lesion is deemed to be necessary. There is a consensus in the literature that surgical intervention in these cases remains a crucial option (Malik et al., 2021[13]; Almazeedi et al, 2022[2]; Yılmaz and Kozan, 2017[20]). Furthermore, a systematic review by Malik et al. demonstrated that surgical management of Dieulafoy's cases was associated with a 100 % success rate, proving the advantage of achieving homeostasis in patients with refractory Dieulafoy's lesions (Malik et al., 2021[13]). Surgical intervention was the definitive treatment for this patient in which the lesion was sutured using prolene 2/0. 

The patient's postoperative course included a five-day stay in the Intensive Therapy Unit (ITU), followed by an uneventful recovery. There were no more episodes of blood loss, and the patient's condition improved. As a result, the patient was discharged from the hospital, indicating that the procedure was successful.

## Conclusions

This case exemplifies a rare cause of upper gastrointestinal bleeding. Surgeons should always maintain a wide range of potential causes, including uncommon ones. While encountering such cases is infrequent, but necessary to provide the accurate diagnosis. Surgeons must be aware that a small, superficial lesion overlying an abnormally large artery can result in life-threatening bleeding which may require large-volume transfusion. Prompt diagnosis and treatment of this case can prevent irreversible complications that can arise. Additionally, in cases of unexplained gastrointestinal bleeding that is recurrent or severe, endoscopy plays a vital role in evaluating and treating such cases while surgery continues as the definitive management of refractory bleeding.

## Declaration

### Acknowledgments

P. Ronan O'Connell: Data collection. P. Ronan O'Connell, Alsalt AL-Busaidi, Jaifar Alomairi, Omer Alabri, Eissa Alwheibi, Alazhar Almaghadari, and Mhmod R. Kadom: Writing and editing of the manuscript; review of the manuscript; final approval of the manuscript; accountable for all aspects of the work.

### Financial interests

None declared.

### Conflict of interest

The authors declare that they have no conflict of interest.

## References

[1] Aletaha N, Hamid H, Yazdi NA, Taslimi R, Shahbazkhani B, Moghadam PK (2019). A rare cause of gastrointestinal bleeding in a 65-year-old man with history of polycythemia vera. Middle East J Dig Dis.

[2] Almazeedi AA, Alkandari MF, Abbas MA, Alsurayei SA, Al-Taher NT (2022). A challenging case of jejunal Dieulafoy’s lesion: a rare cause of refractory lower-gastrointestinal bleeding. Am J Case Rep.

[3] Baettig B, Haecki W, Lammer F, Jost R (1993). Dieulafoy's disease: endoscopic treatment and follow up. Gut.

[4] Cappell MS (2010). Therapeutic endoscopy for acute upper gastrointestinal bleeding. Nat Rev Gastroenterol Hepatol.

[5] Chaptini L, Peikin S, Parrillo JE, Dellinger RP (2008). Gastrointestinal bleeding. Critical care medicine.

[6] Cheng C-L, Liu N-J, Lee C-S, Chen P-C, Ho Y-P, Tang J-H (2004). Endoscopic management of Dieulafoy lesions in acute nonvariceal upper gastrointestinal bleeding. Dig Dis Sci.

[7] Chung I-K, Kim E-J, Lee M-S, Kim H-S, Park S-H, Lee M-H (2000). Bleeding Dieulafoy's lesions and the choice of endoscopic method: comparing the hemostatic efficacy of mechanical and injection methods. Gastrointest Endosc.

[8] El Sayed G, Tarff S, O'Beirne J, Wright G (2015). Endoscopy management algorithms: role of cyanoacrylate glue injection and self-expanding metal stents in acute variceal haemorrhage. Frontline Gastroenterol.

[9] Hamdoun F, Lahmidani N, Lahlali M, Lamine A, Abid H, El Yousfi M (2019). Dieulafoy’s lesion, a challenging diagnosis and therapeutic approach in upper gastrointestinal bleeding. IOSR J Dental Med Sci.

[10] Jeon HK, Kim GH (2015). Endoscopic management of Dieulafoy's lesion. Clin Endosc.

[11] Lara LF, Sreenarasimhaiah J, Tang S-j, Afonso BB, Rockey DC (2010). Dieulafoy lesions of the GI tract: localization and therapeutic outcomes. Dig Dis Sci.

[12] Levy AR, Broad S, Loomis Iii JR, Thomas JA (2022). Diagnosis and treatment of a recurrent bleeding Dieulafoy’s lesion: a case report. Cureus.

[13] Malik A, Inayat F, Goraya MHN, Almas T, Ishtiaq R, Malik S (2021). Jejunal Dieulafoy’s lesion: a systematic review of evaluation, diagnosis, and management. J Investig Med High Impact Case Rep.

[14] Malik TF, Anjum F (2023). Dieulafoys lesion causing gastrointestinal bleeding. StatPearls [Internet].

[15] Nojkov B, Cappell MS (2015). Gastrointestinal bleeding from Dieulafoy’s lesion: clinical presentation, endoscopic findings, and endoscopic therapy. World J Gastrointest Endosc.

[16] Rajanthran SK, Singh HC, Than DJ, Hayati F (2020). Dieulafoy’s lesion: an unexpected and rare cause of upper gastrointestinal bleeding. BMJ Case Rep.

[17] Ribeiro AM, da Silva S, Reis RA, Romero I, Costa S, da Silva JB (2021). Dieulafoy's lesion in the cecum: A rare case report presentation. Int J Surg Case Rep.

[18] Rockey DC (2022). Causes of upper gastrointestinal bleeding in adults: UpToDate. https://www.uptodate.com/contents/causes-of-upper-gastrointestinal-bleeding-in-adults#H6160209.

[19] Yilmaz M, Ozütemiz O, Karasu Z, Ersöz G, Günsar F, Batur Y (2005). Endoscopic injection therapy of bleeding Dieulafoy lesion of the stomach. Hepatogastroenterology.

[20] Yılmaz TU, Kozan R (2017). Duodenal and jejunal Dieulafoy’s lesions: optimal management. Clin Exp Gastroenterol.

